# The genome sequence of the tawny cockroach, Ectobius (Ectobius) pallidus (Olivier, 1789)

**DOI:** 10.12688/wellcomeopenres.23463.1

**Published:** 2025-01-15

**Authors:** Tony Hunter, Natural History Museum Genome Acquisition Lab, Darwin Tree of Life Barcoding collective, Wellcome Sanger Institute Tree of Life Management, Samples and Laboratory team, Wellcome Sanger Institute Scientific Operations: Sequencing Operations, Wellcome Sanger Institute Tree of Life Core Informatics team, Tree of Life Core Informatics collective, Darwin Tree of Life Consortium

**Affiliations:** 1 Entomology Section, World Museum, Liverpool, England, UK

**Keywords:** Ectobius pallidus, spotting Mediterranean cockroach, genome sequence, chromosomal, Blattodea

## Abstract

We present a genome assembly from a specimen of *Ectobius pallidus* (tawny cockroach; Arthropoda; Insecta; Blattodea; Ectobiidae). The assembly contains two haplotypes with total lengths of 2,087.55 megabases and 2,124.67 megabases, respectively. Most of haplotype 1 (98.55%) is scaffolded into 11 chromosomal pseudomolecules, while haplotype 2 is assembled to scaffold level. The mitochondrial genome has also been assembled and is 15.75 kilobases in length.

## Species taxonomy

Eukaryota; Opisthokonta; Metazoa; Eumetazoa; Bilateria; Protostomia; Ecdysozoa; Panarthropoda; Arthropoda; Mandibulata; Pancrustacea; Hexapoda; Insecta; Dicondylia; Pterygota; Neoptera; Polyneoptera; Dictyoptera; Blattodea; Blaberoidea; Ectobiidae; Ectobiinae; *Ectobius*; *Ectobius pallidus* (Olivier, 1789) (NCBI:txid406638)

## Background

*Ectobius* ( *Ectobius*) *pallidus* (Olivier, 1789) is an omnivorous, woodland cockroach. Adults of both sexes are winged and capable of flight. There are six instars and overwintering is undertaken by 4th instar nymphs, before development resumes in April. Adults are present from late June to October/ November. *E. pallidus* has a distinctive golden-brown ground colour with darker brownish spots, and black eyes ( Orthoptera and Allied Insects, 2024).

The species is found throughout Central and Southern Europe (France, UK, Belgium, Spain, Netherlands, Germany, Italy, Luxembourg), and it has been recorded in Algeria and Tunisia ( Cockroach Species File, 2024). It is an introduced species in North America ( GBIF Secretariat, 2024). *E. pallidus* is considered Nationally Scarce in the UK, and Least concern according to the IUCN Red List ( Sutton, 2015). The NBN Atlas shows 866 records for the UK, most of them in southern England and Wales, while it is absent from Scotland ( NBN Atlas Partnership, 2024).

The specimen sequenced here was collected from Hightown, Merseyside, England, and appears to be from the most northerly population in the UK ( NBN Atlas Partnership, 2024). Its geographical isolation on a relatively intensively recorded stretch of coast may suggest a possible introduction. Another local record exists (a morphologically determined nymph from Kirkby, Knowsley, Merseyside, which is around 12 km to the South-East 29.11.1989, Charley Wood, C. Felton, voucher deposited in collections of World Museum).

We present the first genome sequence for *Ectobius pallidus*, collected and sequences as part of the Darwin Tree of Life project ( Blaxter *et al.*, 2022).

## Genome sequence report

**Figure 1.**
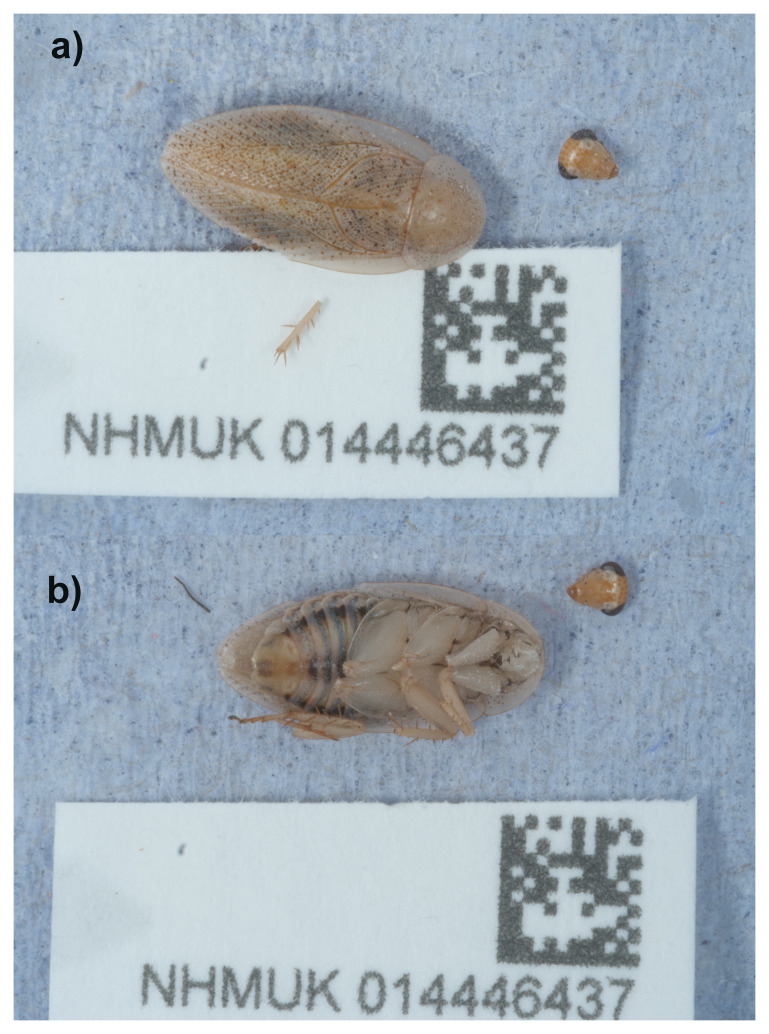
Photographs of the *Ectobius pallidus* (ibEctPall1) specimen used for genome sequencing. **a**) Dorsal view, **b**) Ventral view.

The genome of *Ectobius pallidus* ( Figure 1) was sequenced using Pacific Biosciences single-molecule HiFi long reads, generating a total of 83.72 Gb (gigabases) from 7.83 million reads, providing an estimated 51-fold coverage. Primary assembly contigs were scaffolded with chromosome conformation Hi-C data, which produced 123.13 Gb from 815.42 million reads. Specimen and sequencing details are summarised in Table 1.

**Table 1.** Specimen and sequencing data for *Ectobius pallidus*.

Project information			
**Study title**	Ectobius pallidus		
**Umbrella BioProject**	PRJEB73637		
**Species**	*Ectobius pallidus*		
**BioSample**	SAMEA111458022		
**NCBI taxonomy ID**	406638		
Specimen information			
**Technology**	**ToLID**	**BioSample accession**	**Organism part**
**PacBio long read sequencing**	ibEctPall1	SAMEA111458084	thorax
**Hi-C sequencing**	ibEctPall1	SAMEA111458246	head
Sequencing information			
**Instrument and platform**	**Run accession**	**Read count**	**Base count (Gb)**
**Illumina NovaSeq 6000 (Hi-C)**	ERR12737266	8.15e+08	123.13
**Revio (PacBio)**	ERR12736864	7.83e+06	83.72
**Sequel IIe (PacBio)**	ERR12736865	2.39e+06	27.2

The two haplotypes were combined for curation. Manual assembly curation corrected 147 missing joins or mis-joins and 57 haplotypic duplications. This reduced the assembly length by 1.9% and the scaffold number by 22.24% and increased the scaffold N50 by 7.87%.

**Figure 2.**
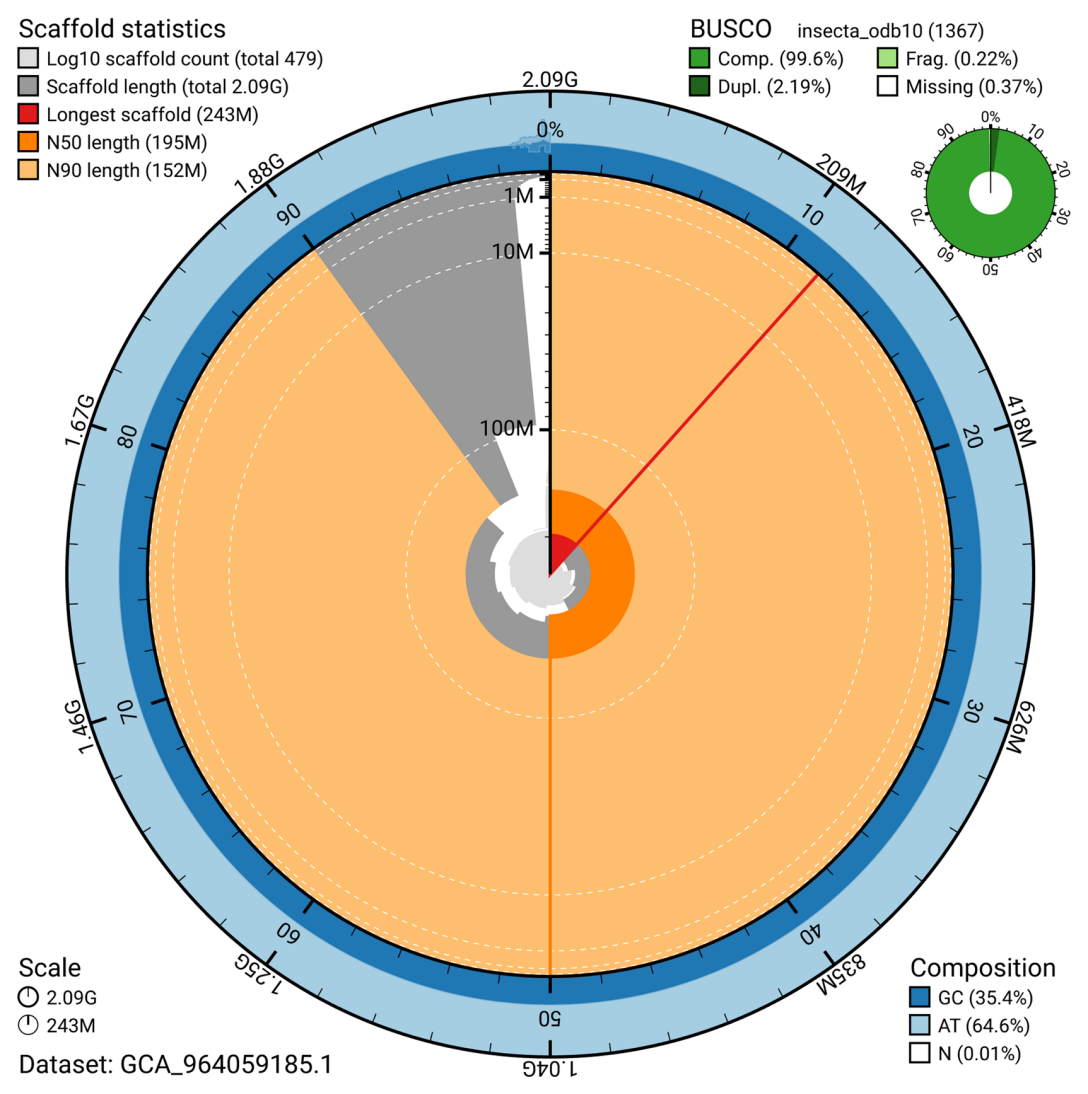
Genome assembly of *Ectobius pallidus*, ibEctPall1.hap1.1: metrics. The BlobToolKit snail plot provides an overview of assembly metrics and BUSCO gene completeness. The circumference represents the length of the whole genome sequence, and the main plot is divided into 1,000 bins around the circumference. The outermost blue tracks display the distribution of GC, AT, and N percentages across the bins. Scaffolds are arranged clockwise from longest to shortest and are depicted in dark grey. The longest scaffold is indicated by the red arc, and the deeper orange and pale orange arcs represent the N50 and N90 lengths. A light grey spiral at the centre shows the cumulative scaffold count on a logarithmic scale. A summary of complete, fragmented, duplicated, and missing BUSCO genes in the insecta_odb10 set is presented at the top right. An interactive version of this figure is available at https://blobtoolkit.genomehubs.org/view/Ectobius_pallidus/dataset/GCA_964059185.1/snail.

**Figure 3.**
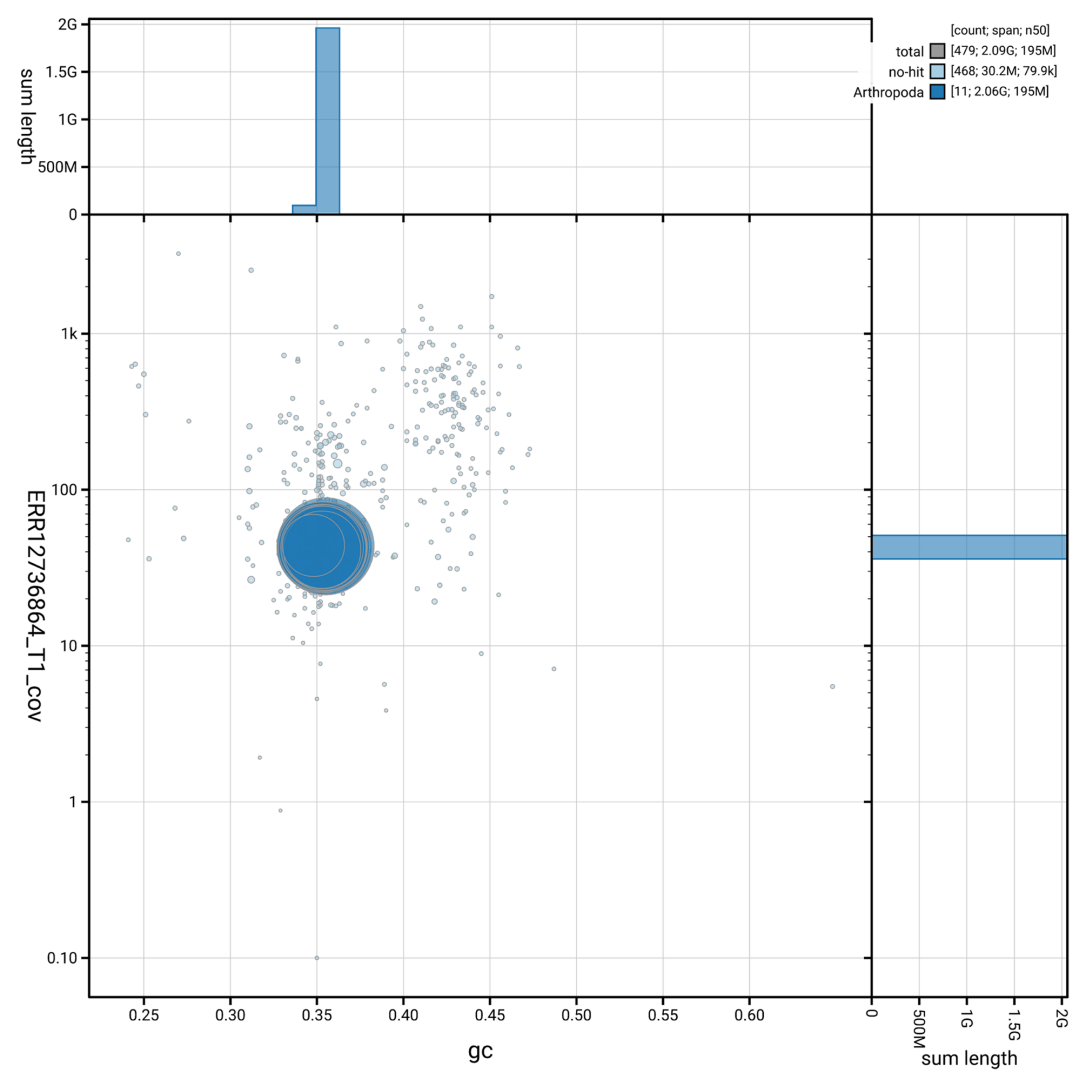
Genome assembly of *Ectobius pallidus*, ibEctPall1.hap1.1: BlobToolKit GC-coverage plot. Blob plot showing sequence coverage (vertical axis) and GC content (horizontal axis). The circles represent scaffolds, with the size proportional to scaffold length and the colour representing phylum membership. The histograms along the axes display the total length of sequences distributed across different levels of coverage and GC content. An interactive version of this figure is available at https://blobtoolkit.genomehubs.org/view/Ectobius_pallidus/dataset/GCA_964059185.1/blob.

**Figure 4.**
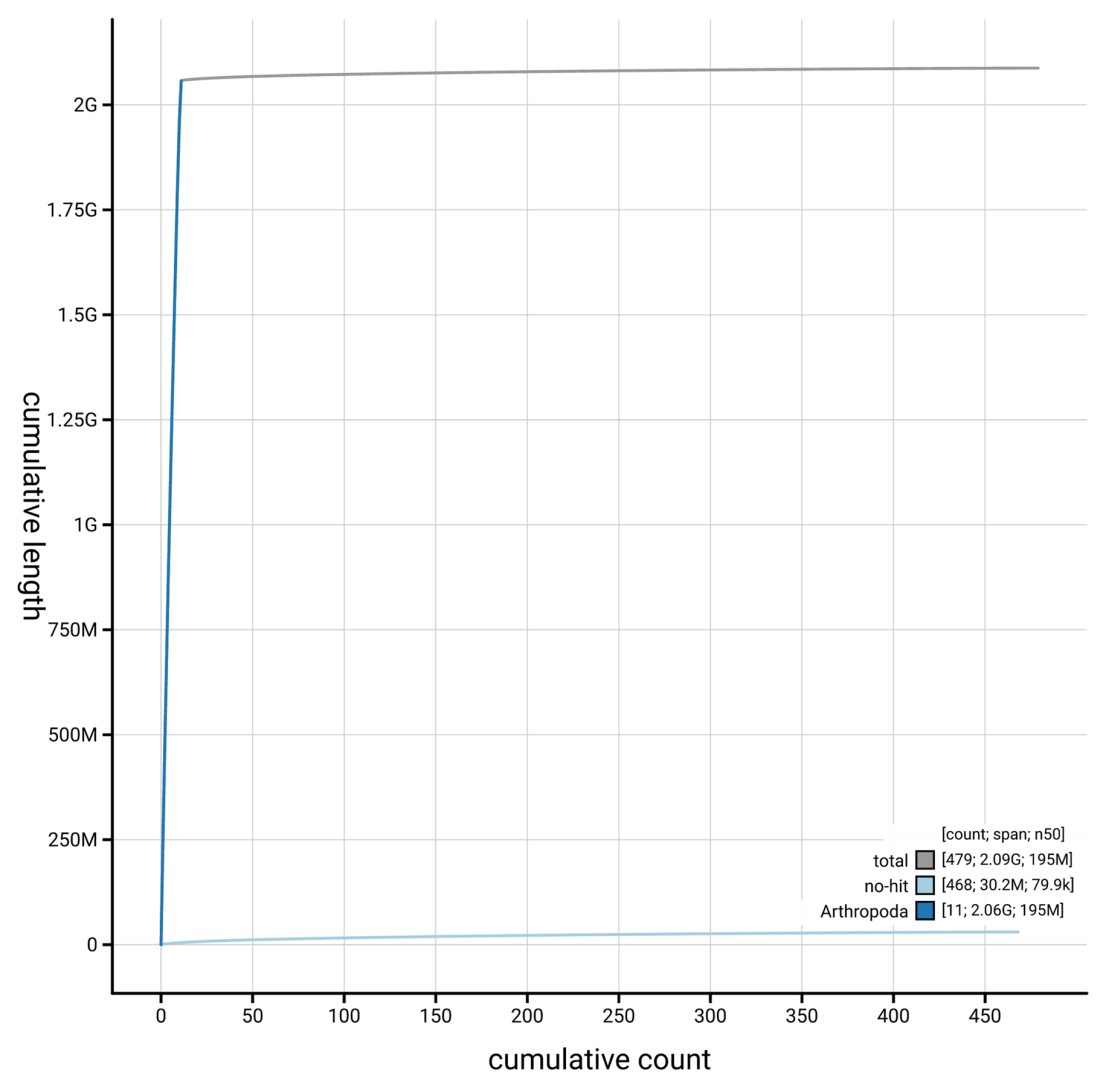
Genome assembly of *Ectobius pallidus* ibEctPall1.hap1.1: BlobToolKit cumulative sequence plot. The grey line shows cumulative length for all scaffolds. Coloured lines show cumulative lengths of scaffolds assigned to each phylum using the buscogenes taxrule. An interactive version of this figure is available at https://blobtoolkit.genomehubs.org/view/Ectobius_pallidus/dataset/GCA_964059185.1/cumulative.

The final haplotype 1 assembly has a total length of 2,087.55 Mb in 478 sequence scaffolds, with 790 gaps, and a scaffold N50 of 194.85 Mb ( Table 2). The snail plot in Figure 2 provides a summary of the assembly statistics, while the distribution of assembly scaffolds on GC proportion and coverage is shown in Figure 3. The cumulative assembly plot in Figure 4 shows curves for subsets of scaffolds assigned to different phyla.

**Table 2.** Genome assembly data for the *Ectobius pallidus* assembly.

Genome assembly	Haplotype 1	Haplotype 2
Assembly name	ibEctPall1.hap1.1	ibEctPall1.hap2.1
Assembly accession	GCA_964059185.1	GCA_964059195.1
Assembly level	chromosome	scaffold
Span (Mb)	2,087.55	2,124.67
Number of contigs	1,268	1,053
Number of scaffolds	478	414
Longest scaffold (Mb)	242.78	None
Assembly metrics for haplotype 1		Benchmark *
Contig N50 length	4.57 Mb	(≥ 1 Mb)
Scaffold N50 length	194.85 Mb	(= chromosome N50)
Consensus quality (QV)	62.9	(≥ 40)
*k*-mer completeness	haplotype 1: 80.46%; haplotype 2: 80.66%; combined: 99.26%	(≥ 95%)
BUSCO **	C:99.6%[S:97.4%,D:2.2%], F:0.2%,M:0.2%,n:1,367	(S > 90%; D < 5%)
Percentage of assembly mapped to chromosomes	98.55%	(≥ 90%)
Sex chromosomes	Not identified	(localised homologous pairs)
Organelles (one complete allele)	Mitochondrial genome: 15.75 kb	

* Assembly metric benchmarks are adapted from Rhie *et al.* (2021) and the Earth BioGenome Project Report on Assembly Standards September 2024. ** BUSCO scores based on the insecta_odb10 BUSCO set using version 5.4.3. C = complete [S = single copy, D = duplicated], F = fragmented, M = missing, n = number of orthologues in comparison.

**Figure 5.**
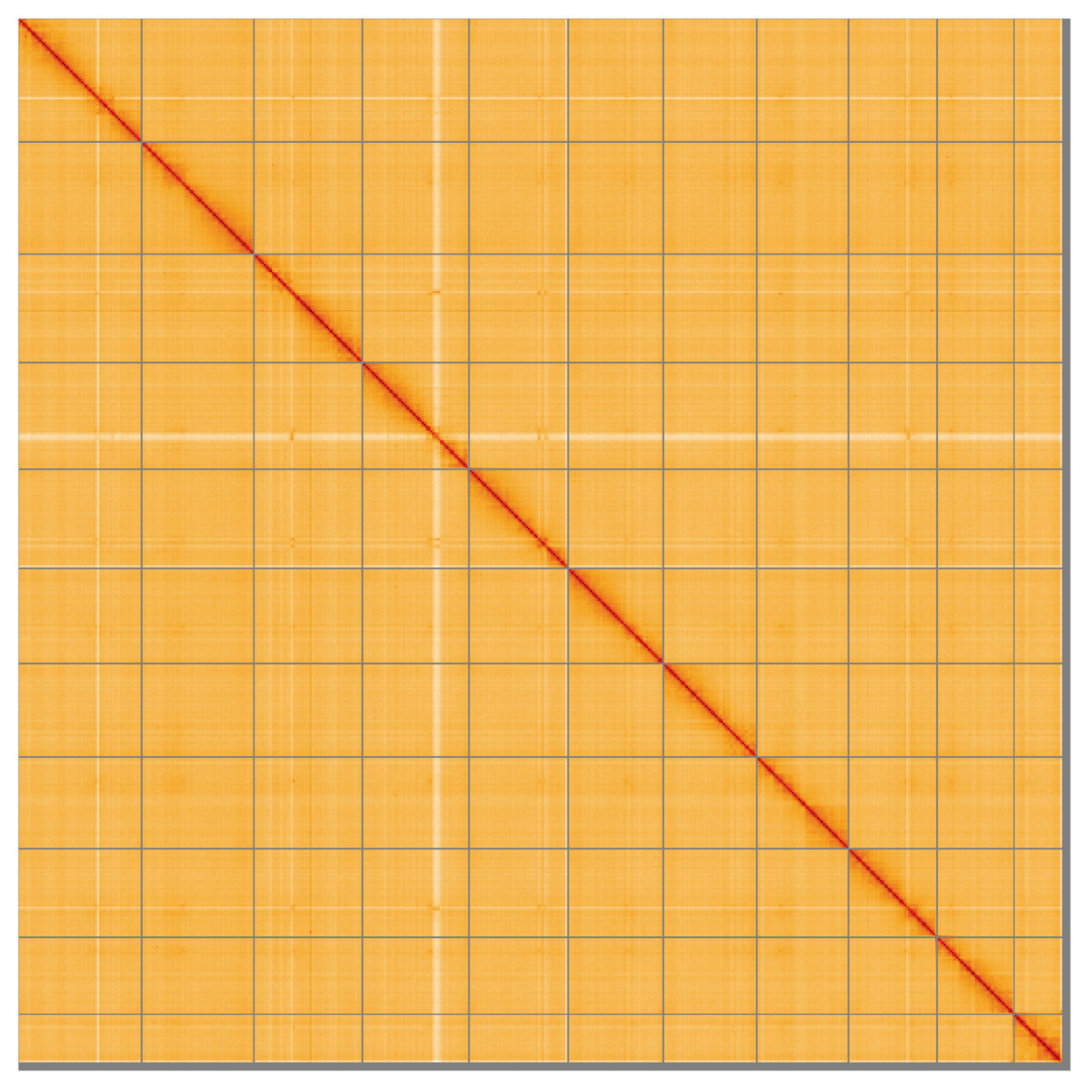
Genome assembly of *Ectobius pallidus* ibEctPall1.hap1.1: Hi-C contact map of the ibEctPall1.hap1.1 assembly, visualised using HiGlass. Chromosomes are shown in order of size from left to right and top to bottom. An interactive version of this figure may be viewed at https://genome-note-higlass.tol.sanger.ac.uk/l/?d=WnP5Ng9kSdqJ2VO821cweA.

Most of the assembly (98.55%) is scaffolded into 11 chromosomal-level scaffolds. Chromosome-scale scaffolds confirmed by the Hi-C data are named in order of size ( Figure 5; Table 3). During manual curation it was noted that chromosome 11 has a heterozygous inversion between 50.1 Mb and 63.8 Mb. The mitochondrial genome was also assembled and is included both as a contig within the multifasta file of the genome submission and as a standalone record in GenBank.

**Table 3.** Chromosomal pseudomolecules in the genome assembly of *Ectobius pallidus*, ibEctPall1.

INSDC accession	Name	Length (Mb)	GC%
OZ060420.1	1	242.78	35.5
OZ060421.1	2	221.55	35.5
OZ060422.1	3	213.8	35.5
OZ060423.1	4	209.59	35.5
OZ060424.1	5	194.85	35.5
OZ060425.1	6	188.25	35.5
OZ060426.1	7	184.15	35.5
OZ060427.1	8	180.63	35.5
OZ060428.1	9	174.2	35.5
OZ060429.1	10	151.56	35.5
OZ060430.1	11	95.97	35
OZ060431.1	MT	0.02	27

The estimated Quality Value (QV) of the final haplotype 1 assembly is 62.9 with *k*-mer completeness of 99.26% for the combined assemblies, and the haplotype 1 assembly has a BUSCO v5.4.3 completeness of 99.6% (single = 97.4%, duplicated = 2.2%), using the insecta_odb10 reference set ( *n* = 1,367).

Metadata for specimens, BOLD barcode results, spectra estimates, sequencing runs, contaminants and pre-curation assembly statistics are given at https://links.tol.sanger.ac.uk/species/406638.

## Methods

### Sample acquisition and DNA barcoding

The specimen submitted to Darwin Tree of Life Project (specimen ID NHMUK014446437, ToLID ibEctPall1) was collected from beneath a wooden board on fixed dunes at Hightown Dunes and Meadows LNR, Sefton, Merseyside (SD296032) on 26.07.2021 by Tony Hunter. The dunes at Hightown are eroding on the seaward side and are also being squeezed against developments inland. *E. pallidus* had also been swept from isolated birch, oak, and willow trees in the same area. Permission to collect on the site was granted by Gordon White (Green Sefton, Sefton Council Ranger Service).

The initial identification was verified by an additional DNA barcoding process according to the framework developed by Twyford *et al*. (2024). A small sample was dissected from the specimens and stored in ethanol, while the remaining parts were shipped on dry ice to the Wellcome Sanger Institute (WSI). The tissue was lysed, the COI marker region was amplified by PCR, and amplicons were sequenced and compared to the BOLD database, confirming the species identification ( Crowley *et al.*, 2023). The barcode was uploaded to BOLD (BOLD id: TPWMB004-21). The barcode was a 100% match to *E. pallidus* specimens from Netherlands, Canada, and over 99% to others from Germany and France. The standard operating procedures for Darwin Tree of Life barcoding have been deposited on protocols.io ( Beasley *et al.*, 2023).

### Nucleic acid extraction

The workflow for high molecular weight (HMW) DNA extraction at the Wellcome Sanger Institute (WSI) Tree of Life Core Laboratory includes a sequence of core procedures: sample preparation and homogenisation, DNA extraction, fragmentation and purification. Detailed protocols are available on protocols.io ( Denton *et al.*, 2023b). The ibEctPall1 sample was prepared for DNA extraction by weighing and dissecting it on dry ice ( Jay *et al.*, 2023), and tissue from the thorax was homogenised using a PowerMasher II tissue disruptor ( Denton *et al.*, 2023a).

HMW DNA was extracted using the Automated MagAttract v1 protocol ( Sheerin *et al.*, 2023). DNA was sheared into an average fragment size of 12–20 kb in a Megaruptor 3 system ( Todorovic *et al.*, 2023). Sheared DNA was purified by solid-phase reversible immobilisation, using AMPure PB beads to eliminate shorter fragments and concentrate the DNA ( Oatley *et al.*, 2023). The concentration of the sheared and purified DNA was assessed using a Nanodrop spectrophotometer and Qubit Fluorometer using the Qubit dsDNA High Sensitivity Assay kit. The fragment size distribution was evaluated by running the sample on the FemtoPulse system.

### Hi-C preparation

Tissue from the head of the ibEctPall1 sample was processed at the WSI Scientific Operations core, using the Arima-HiC v2 kit. Frozen tissue (stored at –80 °C) was fixed, and the DNA crosslinked using a TC buffer with 22% formaldehyde. After crosslinking, the tissue was homogenised using the Diagnocine Power Masher-II and BioMasher-II tubes and pestles. Following the kit manufacturer's instructions, crosslinked DNA was digested using a restriction enzyme master mix. The 5’-overhangs were then filled in and labelled with biotinylated nucleotides and proximally ligated. An overnight incubation was carried out for enzymes to digest remaining proteins and for crosslinks to reverse. A clean up was performed with SPRIselect beads prior to library preparation.

### Library preparation and sequencing

Pacific Biosciences SMRTbell libraries were constructed using the Revio HiFi prep kit, according to the manufacturers’ instructions. DNA sequencing was performed by the Scientific Operations core at the WSI on a Pacific Biosciences Revio instrument.

For Hi-C library preparation, DNA was fragmented to a size of 400 to 600 bp using a Covaris E220 sonicator. The DNA was then enriched, barcoded, and amplified using the NEBNext Ultra II DNA Library Prep Kit following manufacturers’ instructions. The Hi-C sequencing was performed using paired-end sequencing with a read length of 150 bp on an Illumina NovaSeq 6000 instrument.

### Genome assembly, curation and evaluation

***Assembly***

The HiFi reads were first assembled using Hifiasm ( Cheng *et al.*, 2021; Cheng *et al.*, 2022) in Hi-C phasing mode, resulting in a pair of haplotype-resolved assemblies. The Hi-C reads were mapped to the primary contigs using bwa-mem2 ( Vasimuddin *et al.*, 2019). The contigs were further scaffolded using the provided Hi-C data ( Rao *et al.*, 2014) in YaHS ( Zhou *et al.*, 2023) using the --break option for handling potential misassemblies. The scaffolded assemblies were evaluated using Gfastats ( Formenti *et al.*, 2022), BUSCO ( Manni *et al.*, 2021) and MERQURY.FK ( Rhie *et al.*, 2020).

The mitochondrial genome was assembled using MitoHiFi ( Uliano-Silva *et al.*, 2023), which runs MitoFinder ( Allio *et al.*, 2020) and uses these annotations to select the final mitochondrial contig and to ensure the general quality of the sequence.

***Assembly curation***

The assembly was decontaminated using the Assembly Screen for Cobionts and Contaminants (ASCC) pipeline (article in preparation). Flat files and maps used in curation were generated in TreeVal ( Pointon *et al.*, 2023). Manual curation was primarily conducted using PretextView ( Harry, 2022), with additional insights provided by JBrowse2 ( Diesh *et al.*, 2023) and HiGlass ( Kerpedjiev *et al.*, 2018). Scaffolds were visually inspected and corrected as described by Howe *et al*. (2021). Any identified contamination, missed joins, and mis-joins were corrected, and duplicate sequences were tagged and removed. The curation process is documented at https://gitlab.com/wtsi-grit/rapid-curation (article in preparation).

***Evaluation of the final assembly***

The final assembly was post-processed and evaluated using the three Nextflow ( Di Tommaso *et al.*, 2017) DSL2 pipelines: sanger-tol/readmapping ( Surana *et al.*, 2023a), sanger-tol/genomenote ( Surana *et al.*, 2023b), and sanger-tol/blobtoolkit ( Muffato *et al.*, 2024). The readmapping pipeline aligns the Hi-C reads using bwa-mem2 ( Vasimuddin *et al.*, 2019) and combines the alignment files with SAMtools ( Danecek *et al.*, 2021). The genomenote pipeline converts the Hi-C alignments into a contact map using BEDTools ( Quinlan & Hall, 2010) and the Cooler tool suite ( Abdennur & Mirny, 2020). The contact map is visualised in HiGlass ( Kerpedjiev *et al.*, 2018). This pipeline also computes *k*-mer completeness and QV consensus quality values with FastK and MERQURY.FK, and runs BUSCO ( Manni *et al.*, 2021) to assess completeness.

The blobtoolkit pipeline is a Nextflow port of the previous Snakemake Blobtoolkit pipeline ( Challis *et al.*, 2020). It aligns the PacBio reads in SAMtools and minimap2 ( Li, 2018) and generates coverage tracks for regions of fixed size. In parallel, it queries the GoaT database ( Challis *et al.*, 2023) to identify all matching BUSCO lineages to run BUSCO ( Manni *et al.*, 2021). For the three domain-level BUSCO lineages, the pipeline aligns the BUSCO genes to the UniProt Reference Proteomes database ( Bateman *et al.*, 2023) with DIAMOND ( Buchfink *et al.*, 2021) blastp. The genome is also split into chunks according to the density of the BUSCO genes from the closest taxonomic lineage, and each chunk is aligned to the UniProt Reference Proteomes database with DIAMOND blastx. Genome sequences without a hit are chunked with seqtk and aligned to the NT database with blastn ( Altschul *et al.*, 1990). The blobtools suite combines all these outputs into a blobdir for visualisation.

The genome evaluation pipelines were developed using nf-core tooling ( Ewels *et al.*, 2020) and MultiQC ( Ewels *et al.*, 2016), relying on the Conda package manager, the Bioconda initiative ( Grüning *et al.*, 2018), the Biocontainers infrastructure ( da Veiga Leprevost *et al.*, 2017), as well as the Docker ( Merkel, 2014) and Singularity ( Kurtzer *et al.*, 2017) containerisation solutions.

Table 4 contains a list of relevant software tool versions and sources.

**Table 4.** Software tools: versions and sources.

Software tool	Version	Source
BEDTools	2.30.0	https://github.com/arq5x/bedtools2
BLAST	2.14.0	ftp://ftp.ncbi.nlm.nih.gov/blast/executables/blast+/
BlobToolKit	4.3.7	https://github.com/blobtoolkit/blobtoolkit
BUSCO	5.4.3 and 5.5.0	https://gitlab.com/ezlab/busco
bwa-mem2	2.2.1	https://github.com/bwa-mem2/bwa-mem2
Cooler	0.8.11	https://github.com/open2c/cooler
DIAMOND	2.1.8	https://github.com/bbuchfink/diamond
fasta_windows	0.2.4	https://github.com/tolkit/fasta_windows
FastK	427104ea91c78c3b8b8b49f1a7d6bbeaa869ba1c	https://github.com/thegenemyers/FASTK
Gfastats	1.3.6	https://github.com/vgl-hub/gfastats
GoaT CLI	0.2.5	https://github.com/genomehubs/goat-cli
Hifiasm	0.19.8-r603	https://github.com/chhylp123/hifiasm
HiGlass	44086069ee7d4d3f6f3f0012569789ec138f42b84a a44357826c0b6753eb28de	https://github.com/higlass/higlass
Merqury.FK	d00d98157618f4e8d1a9190026b19b471055b22e	https://github.com/thegenemyers/MERQURY.FK
MitoHiFi	3	https://github.com/marcelauliano/MitoHiFi
MultiQC	1.14, 1.17, and 1.18	https://github.com/MultiQC/MultiQC
NCBI Datasets	15.12.0	https://github.com/ncbi/datasets
Nextflow	23.04.0-5857	https://github.com/nextflow-io/nextflow
PretextView	0.2	https://github.com/sanger-tol/PretextView
purge_dups	1.2.5	https://github.com/dfguan/purge_dups
samtools	1.16.1, 1.17, and 1.18	https://github.com/samtools/samtools
sanger-tol/ascc	-	https://github.com/sanger-tol/ascc
sanger-tol/blobtoolkit	0.6.0	https://github.com/sanger-tol/blobtoolkit
sanger-tol/genomenote	1.2.2.	https://github.com/sanger-tol/genomenote
sanger-tol/readmapping	1.2.1	https://github.com/sanger-tol/readmapping
Seqtk	1.3	https://github.com/lh3/seqtk
Singularity	3.9.0	https://github.com/sylabs/singularity
TreeVal	1.0.0	https://github.com/sanger-tol/treeval
YaHS	1.2a.2	https://github.com/c-zhou/yahs

### Wellcome sanger institute – legal and governance

The materials that have contributed to this genome note have been supplied by a Darwin Tree of Life Partner. The submission of materials by a Darwin Tree of Life Partner is subject to the **‘Darwin Tree of Life Project Sampling Code of Practice’**, which can be found in full on the Darwin Tree of Life website here. By agreeing with and signing up to the Sampling Code of Practice, the Darwin Tree of Life Partner agrees they will meet the legal and ethical requirements and standards set out within this document in respect of all samples acquired for, and supplied to, the Darwin Tree of Life Project.

Further, the Wellcome Sanger Institute employs a process whereby due diligence is carried out proportionate to the nature of the materials themselves, and the circumstances under which they have been/are to be collected and provided for use. The purpose of this is to address and mitigate any potential legal and/or ethical implications of receipt and use of the materials as part of the research project, and to ensure that in doing so we align with best practice wherever possible. The overarching areas of consideration are:

•   Ethical review of provenance and sourcing of the material

•   Legality of collection, transfer and use (national and international)

Each transfer of samples is further undertaken according to a Research Collaboration Agreement or Material Transfer Agreement entered into by the Darwin Tree of Life Partner, Genome Research Limited (operating as the Wellcome Sanger Institute), and in some circumstances other Darwin Tree of Life collaborators.

## Data Availability

European Nucleotide Archive: Ectobius pallidus. Accession number PRJEB73637; https://identifiers.org/ena.embl/PRJEB73637. The genome sequence is released openly for reuse. The *Ectobius pallidus* genome sequencing initiative is part of the Darwin Tree of Life (DToL) project. All raw sequence data and the assembly have been deposited in INSDC databases. The genome will be annotated using available RNA-Seq data and presented through the Ensembl pipeline at the European Bioinformatics Institute. Raw data and assembly accession identifiers are reported in Table 1 and Table 2.

## Author information

Members of the Natural History Museum Genome Acquisition Lab are listed here: https://doi.org/10.5281/zenodo.12159242.

Members of the Darwin Tree of Life Barcoding collective are listed here: https://doi.org/10.5281/zenodo.12158331.

Members of the Wellcome Sanger Institute Tree of Life Management, Samples and Laboratory team are listed here: https://doi.org/10.5281/zenodo.12162482.

Members of Wellcome Sanger Institute Scientific Operations: Sequencing Operations are listed here: https://doi.org/10.5281/zenodo.12165051.

Members of the Wellcome Sanger Institute Tree of Life Core Informatics team are listed here: https://doi.org/10.5281/zenodo.12160324.

Members of the Tree of Life Core Informatics collective are listed here: https://doi.org/10.5281/zenodo.12205391.

Members of the Darwin Tree of Life Consortium are listed here: https://doi.org/10.5281/zenodo.4783558.
