# Supplementary material for: The genome sequence of the tawny cockroach, Ectobius (Ectobius) pallidus (Olivier, 1789)
Source: Wellcome Open Res. 2025 Jan 15;10:22. [Version 1] doi: 10.12688/wellcomeopenres.23463.1 (PMC11754957; doi:10.12688/wellcomeopenres.23463.1)
Supplement: Peer review history

# Reviewer report: Reviewer response for version 1

**DOI:** 10.21956/wellcomeopenres.25873.r117416

**Published:** 2025-02-04

**Authors:** Terrence Sylvester

**Affiliations:** 1 University of Memphis, Memphis, TN, USA

The author presents the genome of the tawny cockroach, *Ectobius pallidus*, which is scaffolded up to chromosome scale. The author identifies 11 chromosomal pseudomolecules in the genome. The author does mention that they used the primary contigs for scaffolding. The assembly program hifiasm used in Hi-C phasing mode produces a primary assembly and an assembly for each haplotype. Primary assembly is not phased, meaning it does not represent a single haplotype. Instead, it is a mosaic of all the haplotypes. So, when the authors mentioned that they assembled and scaffolded the two haplotypes in the abstract, the methods do not fully convey that is what the authors have done. Despite that, the genome presented here appears highly contiguous and largely complete (regarding BUSCO completeness). The author does not mention to what degree haplotype two was scaffolded (e.g. statistics like number of contigs, N50, BUSCO comparing the two haplotypes would be sufficient).

Are sufficient details of methods and materials provided to allow replication by others?

Yes

Is the rationale for creating the dataset(s) clearly described?

Yes

Are the datasets clearly presented in a useable and accessible format?

Yes

Are the protocols appropriate and is the work technically sound?

Yes

Reviewer Expertise:

Genomics

I confirm that I have read this submission and believe that I have an appropriate level of expertise to confirm that it is of an acceptable scientific standard.

# Reviewer report: Reviewer response for version 1

**DOI:** 10.21956/wellcomeopenres.25873.r117418

**Published:** 2025-01-22

**Authors:** Ayşe Nur Pektaş

**Affiliations:** 1 Advanced Technology Research Center (CUTAM), Sivas Cumhuriyet University, Sivas, Turkey

The authors and the project team assembled a genome from a sample of *Ectobius pallidus* (Insecta; Blattodea; Ectobiidae). In this study, the authors showed that the genome assembly contained two haplotypes and also assembled and displayed the mitochondrial genome.

 This study is interesting and very informative because it provides detailed information about the working method (especially the assembly and curation sections).

 The accessibility of the study data is very useful. I congratulate them for providing a nice resource for genome assembly research.

Are sufficient details of methods and materials provided to allow replication by others?

Yes

Is the rationale for creating the dataset(s) clearly described?

Yes

Are the datasets clearly presented in a useable and accessible format?

Yes

Are the protocols appropriate and is the work technically sound?

Yes

Reviewer Expertise:

insect phylogeny, insects DNA barcoding, Cockroach DNA barcoding, Insect antimicrobial peptides, virology,

I confirm that I have read this submission and believe that I have an appropriate level of expertise to confirm that it is of an acceptable scientific standard.

# Reviewer report: Reviewer response for version 1

**DOI:** 10.21956/wellcomeopenres.25873.r117412

**Published:** 2025-01-18

**Authors:** Dominic A Evangelista

**Affiliations:** 1 University of Illinois Chicago, Chicago, Illinois, USA

The report gives descriptive statistics for a newly sequenced genome of *E. pallidus*. As an evolutionarily distinct lineage with modest species richness, it is useful to have a full genome for Ectobiidae, especially one with such high completeness. This data is fundamentally needed and it is presented here well. 

 My suggestions on how to improve the report are as follows:

 More context can be given in the background section. There are other published genomes and genome-scale data (transcriptomes) for Blattodea. A little more about them would assist in setting appropriate context. Also, it's good that some description of *Ectobius pallidu*s' ecology is given but there is other relevant information for genomes (e.g., evolutionary context, physiological aspects of interests). So, I think 3 or more additional short paragraphs of context would be justified.

 Fig 1 could be much more effective. I would suggest that the author refine the figure so it emphasizes it's goal. If the goal is to act as a visual voucher, then it would be much more effective to show the insect at greater magnification, so the reader can see details. I think one of the functions it serves is to verify that the specimen is correctly identified. Also, the label need not be shown twice, and can be cropped and shown separately from the insect.

 Fig 3 Is there a way to rescale the elements here? It might be better to log transform circle sizes or to color them by scaffold length instead of taxonomic affiliation. You can just give the taxonomic affiliation stats in the text. But maybe there is a trend being shown with the current layout, but it's hard to see because the small circles are too small and the colors are too similar to differentiate.

 Regarding the "phylum membership stats", most (all?) of the short scaffolds are "no-hit". The text should explain why this is (e.g., mobile genetic elements?, BLAST database incompleteness?).

 Fig 4. My understanding of this figure is that it separates the scaffolds by their taxon affiliation, and demonstrates that the Arthropoda ones are the majority of bps. If there is something else to get from this I would suggest modifying the caption. 

 Fig. 5. More needs to be explained in the caption. Explain what the colors mean and that the blue lines delimit the chromosomes.

Are sufficient details of methods and materials provided to allow replication by others?

Yes

Is the rationale for creating the dataset(s) clearly described?

Partly

Are the datasets clearly presented in a useable and accessible format?

Yes

Are the protocols appropriate and is the work technically sound?

Yes

Reviewer Expertise:

Phylogenomics, cockroach biodiversity

I confirm that I have read this submission and believe that I have an appropriate level of expertise to confirm that it is of an acceptable scientific standard.
